# Blood Lactate/ATP Ratio, as an Alarm Index and Real-Time Biomarker in Critical Illness

**DOI:** 10.1371/journal.pone.0060561

**Published:** 2013-04-05

**Authors:** Junji Chida, Rie Ono, Kazuhiko Yamane, Mineyoshi Hiyoshi, Masaji Nishimura, Mutsuo Onodera, Emiko Nakataki, Koichi Shichijo, Masatami Matushita, Hiroshi Kido

**Affiliations:** 1 Division of Enzyme Chemistry, Institute for Enzyme Research, The University of Tokushima, Tokushima, Japan; 2 Emergency and Critical Care Unit, Tokushima University Hospital, The University of Tokushima, Tokushima, Japan; 3 Department of Pediatrics, Tokushima Red Cross Hospital, Tokushima, Japan; St. Joseph’s Hospital and Medical Center, United States of America

## Abstract

**Objective:**

The acute physiology, age and chronic health evaluation (APACHE) II score and other related scores have been used for evaluation of illness severity in the intensive care unit (ICU), but there is still a need for real-time and sensitive prognostic biomarkers. Recently, alarmins from damaged tissues have been reported as alarm-signaling molecules. Although ATP is a member of the alarmins and its depletion in tissues closely correlates with multiple-organ failure, blood ATP level has not been evaluated in critical illness. To identify real-time prognostic biomarker of critical illness, we measured blood ATP levels and the lactate/ATP ratio (ATP-lactate energy risk score, A-LES) in critically ill patients.

**Methods and Results:**

Blood samples were collected from 42 consecutive critically ill ICU patients and 155 healthy subjects. The prognostic values of blood ATP levels and A-LES were compared with APACHE II score. The mean ATP level (SD) in healthy subjects was 0.62 (0.19) mM with no significant age or gender differences. The median ATP level in severely ill patients at ICU admission was significantly low at 0.31 mM (interquartile range 0.25 to 0.44) than the level in moderately ill patient at 0.56 mM (0.38 to 0.70) (*P*<0.01). Assessment with ATP was further corrected by lactate and expressed as A-LES. The median A-LES was 2.7 (2.1 to 3.3) in patients with satisfactory outcome at discharge but was significantly higher in non-survivors at 38.9 (21.0 to 67.9) (*P*<0.01). Receiver operating characteristic analysis indicated that measurement of blood ATP and A-LES at ICU admission are as useful as APACHE II score for prediction of mortality.

**Conclusion:**

Blood ATP levels and A-LES are sensitive prognostic biomarkers of mortality at ICU admission. In addition, A-LES provided further real-time evaluation score of illness severity during ICU stay particularly for critically ill patients with APACHE II scores of ≥20.0.

## Introduction

The recent years have witnessed a wide used of the acute physiology, age and chronic health evaluation (APACHE) II score [1], the simplified acute physiology score (SAPS) II [2] and other related scoring systems [3], [4] in the evaluation of the severity of illness in the intensive care unit (ICU). These scoring systems have several drawbacks, in particular their time-consuming evaluation, some unavailable data from automatic equipments and inter-examiner discrepancies [5]. Thus, these scores are not always utilized in daily practice of many ICUs [6].

Adenosine 5′-triphosphate (ATP) is the “energy currency” of organisms and plays central roles in bioenergetics, whereby its level is used to evaluate cell viability and proliferation [7]–[10], cell death [11], [12], and energy transmission [13]. Human typically uses about body weight of ATP over the course of the day [14]. In addition, ATP release from damaged cells and tissues has recently attracted attention, and has been reported as an alarm signal compound, alarmin [15], [16]. Alarmins were originally categorized as endogenous damage-associated molecular pattern (DAMP) molecules, to separate them from exogenous pathogen-associated molecular pattern (PAMP) molecules [17], associated with overstimulation of the immune system [18]. The released ATP in serum, however, is rapidly degraded within few minutes [19] and the levels are difficult to evaluate correctly. Therefore, we can only monitor ATP levels in blood cells, as the net value of intracellular ATP production and ATP degradation and/or release.

The three main pathways to generate ATP in eukaryotic organisms are glycolysis, the citric acid cycle/oxidative phosphorylation and fatty acid β-oxidation. Once ATP generation in the mitochondria is impaired in various diseases, energy source metabolites, such as carbohydrate metabolites and fatty acid metabolites are converted to and stored as lactate and ketone bodies, respectively. In fact, hyperlactatemia develops in nearly half of patients admitted to the ICU, and presentation with or development of hyperlactatemia is associated with a significant increase in the incidence of organ failure, metabolic dysfunction and mortality [20]. To find real-time and reliable biomarker(s) for the progression state of critical illness, we evaluated blood ATP levels in combination with serum lactate levels as a new alarm reporter in critical illness and the values were compared with the APACHE II score.

Critically ill patients with multiple organ failure (MOF) and septic non-survivors show a decrease in mitochondrial activity and ATP production, and increase in lactate concentrations in leg muscles [21]–[23]. In addition, we recently demonstrated that influenza A virus infection triggers MOF and acute myocarditis with ATP depletion in mice as well as impairment of mitochondrial membrane potential in cardiomyoblasts [24]. Blood ATP depletion was also identified in children with influenza-associated acute encephalopathy and in patients with mitochondrial diseases [25].

In the measurement of ATP levels in various tissues and blood, we recently found that the chaotropic ATP extraction reagents recommended in the commercially available assay kits so far (e.g., trichloroacetic acid, perchloric acid and ethylene glycol), are useful only for materials with relatively low protein concentrations, but not suitable for tissues with high protein concentrations. ATP is co-precipitated with insoluble protein during homogenization in high protein concentrations. Accordingly, we improved the ATP extraction efficiency from tissues and cells using a novel phenol-based extraction reagent [26].

The present study was designed to determine the control gender-specific blood ATP levels measured in healthy individuals of various ages, using our highly reliable extraction method. We then used the “normal” blood ATP levels of the control to evaluate the blood ATP levels and lactate (mM)/ATP (mM) ratio (expressed as the ATP-lactate energy risk score, A-LES) in patients admitted to the ICU. The A-LES was used as a real-time prognostic alarm biomarker and the values were compared with the APACHE II scores.

## Materials and Methods

### Ethics Statement

For clinical studies, written informed consent was obtained directly from each study participant or their legal representative before enrolment. Also, all healthy individuals gave assent if able to understand, and their parents or guardians gave written informed consent and permission to participate in this study. Permission to perform scientific studies and ethical approval of the study protocol were granted the Ethics Committee of Tokushima University Hospital (Permit Number: #901). The study was conducted under the supervision of the physicians involved (MN, RO, MO, EN, KS and MM), and patients were advised of risks, benefits and the right to withdraw from further involvement in the study at any point without repercussions. All data, particularly patient identifying data, were physically and electronically secured throughout the study.

### Patients

We evaluated 42 consecutive patients admitted to ICU from November 2009 to November 2010. All patients received early goal-directed therapy according to a standard protocol that emphasized adequate volume administration, appropriate therapeutic drug administration, and optimal oxygen delivery. The study also included 155 healthy individuals free from any acute or chronic illness.

### Blood Collection

Blood was withdrawn from the antecubital vein of healthy individuals into either Vacutainer tubes (BD vacutainer; Becton Dickinson Diagnostics, Tokyo, Japan) or syringes containing either ethylenediaminetetraacetic acid (EDTA) or sodium heparin. For ICU patients, the blood samples were usually collected from the arterial line into EDTA vacutainers, but in some cases collected from the antecubital vein or central vein. In each patient, blood was sampled at serial time points during the ICU stay. After withdrawal of 5.0 mL of arterial/venous blood, the sample was transferred to a 15.0-mL Falcon tube. Blood gas data, such as PO_2_ and PCO_2_, and data of total hemoglobin (tHb), blood glucose (BG), and lactate were monitored by a blood gas analyzer (Blood Gas System 860; Bayer Diagnostics, Tokyo, Japan). Blood aliquots (0.1 mL) were added to 1.3 mL of Tris-EDTA-saturated phenol (phenol-TE) ATP extraction reagents (AMERIC-ATP kit; Wako Pure Chemical Industries, Osaka, Japan), thoroughly shaken for 20 seconds and then stored at −20°C until use.

### Measurement of Blood ATP

Blood ATP levels were measured by the firefly bioluminescence assay kit (AMERIC-ATP kit; Wako Pure Chemical Industries, Osaka, Japan) according to the protocol supplied by the manufacturer or as described previously [26]. Briefly, the extracted blood sample was shaken and centrifuged (10,000 × *g*, 5 minutes at 4°C) to achieve phase separation; 50 µL of the upper aqueous phases was diluted 10,000-fold with deionized water. Then 10 µL of this diluted extract was injected into 90 µL of luciferin/luciferase mixture, and the bioluminescence product was immediately measured by a luminometer (GloMax-96 Microplate Luminometer; Promega, Tokyo, Japan). Blood ATP level (mM) in each sample was calculated from the calibration curve.

### Calculation of APACHE II Score and A-LES

The severity of illness was evaluated in each patient within the first 24 hours of ICU admission using the APACHE II score [1], [27]. The score was also determined every 24 hours during the ICU stay. The A-LES score, representing [serum lactate (mM)/blood ATP (mM)], was determined for each patient during the ICU stay.

### Statistical Analysis

ICU patients were divided into two groups based on the severity of illness on admission: moderately ill patients (APACHE II score <20) and severely ill patients (APACHE II score ≥20). The outcome of the patients was divided into two categories: survival and non-survival. Data were analyzed for statistical significance across groups using nonparametric Mann-Whitney’s U test. Correlations were calculated by determining Spearman’s rank correlation coefficient (*r_s_*). *P* values less than 0.05 were considered statistically significant. Receiver operating characteristic (ROC) curves were constructed using Microsoft Excel software (Microsoft Corporation, Redmond, WA) add-in Ekuseru-Toukei 2010 version 1.10 (Social Survey, Research Information Co.) to evaluate the accuracy of risk prediction comparing the calculated mortality with the actual deaths.

## Results

### ATP, Lactate and A-LES Values in Healthy Subjects

At the beginning of the study, we determined the levels of ATP by the new phenol-TE extraction method [26] and lactate in venous blood samples from 155 healthy males and females (age, range 0 to 92 years). The measured levels showed normal distribution pattern, with a mean (SD) value of 0.62 mM (0.19) (Table 1). There was no significant sex difference in ATP level. The ATP levels tended to be slightly lower in subjects aged ≥60 years than those in younger subjects, although the difference was not significant. The mean blood lactate level under resting-state condition was <1.19 mM (<0.50) in the healthy group, with no significant age or gender difference. The mean A-LES (SD) was <2.00 (0.83) with no significant age or gender difference in the control subjects.

**Table 1:** Whole blood ATP, lactate and A-LES levels in healthy subjects.

Age (years)	Sex	Lactate (mM)	ATP (mM)	A-LES
0 to 19	Males (*n = 6*)	1.43±1.07	0.71±0.11	1.50±1.46
	Females (*n = 7*)	<1.69±1.06	0.64±0.13	<2.88±1.70
20 to 29	Males (*n = 11*)	1.48±0.38	0.79±0.18	1.99±0.61
	Females (*n = 10*)	1.03±0.25	0.71±0.20	1.40±0.48
30 to 39	Males (*n = 15*)	<1.38±0.47	0.68±0.11	<2.05±0.84
	Females (*n = 8*)	1.20±0.25	0.79±0.19	1.60±0.47
40 to 49	Males (*n = 12*)	1.46±0.39	0.83±0.25	1.90±0.73
	Females (*n = 12*)	<1.05±0.35	0.67±0.17	<1.63±0.60
50 to 59	Males (*n = 9*)	<1.38±0.48	0.64±0.19	<2.17±0.45
	Females (*n = 4*)	<0.81±0.03	0.52±0.12	<1.61±0.32
60 to 69	Males (*n = 8*)	<1.05±0.27	0.52±0.10	<2.04±0.54
	Females (*n = 11*)	<0.81±0.03	0.47±0.05	<1.73±0.18
70 to 92	Males (*n = 13*)	<0.99±0.23	0.47±0.06	<2.14±0.62
	Females (*n = 29*)	<1.00±0.36	0.46±0.05	<2.18±0.73
0 to 92	Males (*n = 74*)	<1.31±0.49	0.66±0.20	<2.05±0.73
	Females (*n = 81*)	<1.08±0.49	0.57±0.17	<1.96±0.91
	Total (*n = 155*)	<1.19±0.50	0.62±0.19	<2.00±0.83

Data are mean±SD. *n* = number of healthy subjects. Blood lactate levels below the limit of measurement (<0.8 mM) are reported as <0.8 mM.

### Similar ATP and Lactate Levels in Arterial and Central Venous Blood

Table 2 shows the data of ATP, lactate and A-LES levels in arterial and central venous blood of 7 representative ICU patients. Regression analysis to validate the correlation among ATP, lactate and A-LES levels in arterial and central venous blood showed almost perfect correlation with high correlation coefficients: *r_s_* = 1.00 for ATP, *r_s_* = 0.98 for lactate and *r_s_* = 1.00 for A-LES between blood collected from radial and pulmonary arteries (*P*<0.001); and *r_s_* = 1.00 for ATP, *r_s_* = 0.97 for lactate and *r_s_* = 0.99 for A-LES between radial artery and central venous blood (*P*<0.001). The results were consistent with the previous report of equivalent lactate levels in blood samples from peripheral vein, pulmonary artery and central vein [28].

**Table 2:** Comparison of blood lactate and ATP levels in radial arterial (A), pulmonary arterial (PA), and central venous (CV) blood.

Patients no./Time (h)*	Lactate (mM)			ATP (mM)			A-LES		
	A	PA	CV	A	PA	CV	A	PA	CV
07/0	1.79	1.69	1.97	0.70	0.72	0.73	2.56	2.35	2.70
07/3	1.95	1.97	2.16	0.83	0.83	0.80	2.35	2.37	2.70
07/6	2.09	2.01	2.01	0.34	0.35	0.34	6.15	5.74	5.91
07/24	1.68	1.69	1.63	2.88	2.88	2.76	2.47	2.49	2.60
08/3	2.88	2.88	2.76	0.96	0.99	0.98	3.00	2.91	2.82
08/6	2.47	2.49	2.60	0.55	0.53	0.55	4.49	4.70	4.73
09/0	2.29	2.43	2.73	0.36	0.40	0.37	6.36	6.08	7.38
10/3	1.64	–	1.51	0.33	–	0.34	4.97	–	4.44
18/0	6.10	5.48	5.75	0.42	0.41	0.41	14.52	13.37	14.02
19/0	5.39	5.72	5.91	0.35	0.38	0.38	15.40	15.03	15.55
21/0	1.47	1.30	1.37	0.53	0.57	0.53	2.77	2.28	2.58

* Time period (h, hours) after ICU admission.

### ATP, Lactate Levels and A-LES in ICU Patients

Next, we measured the levels of blood ATP and serum lactate and calculated the A-LES in 42 patients admitted to the ICU. The levels of these parameters showed skewed distribution. Table 3 lists the demographic data while Tables S1, S2 and S3 list the individual data and clinical characteristics. The major diagnosis on admission was post-cardiovascular surgery (21.4%), followed by septic shock (16.7%). The median values (interquartile range) of ATP, lactate and A-LES were 0.38 mM (0.29 to 0.56), 2.72 mM (1.92 to 5.78 mM) and 8.00 (4.74 to 13.37), respectively, and the levels were all within the abnormal range compared to those in healthy subjects (Table 1).

**Table 3:** Patient demographics and clinical findings.

Patient demographics	
n	42
Age, years	68 (34 to 79)
Sex, % male:% female	52∶48
First 24-h APACHE II score	18 (13 to 25)
Duration of ICU stay, days	3 (1 to 10)
Diagnosis at admission	No. of patients (%)
Post-cardiovascular surgery	9 (21.4)
Septic shock	7 (16.7)
Acute coronary syndrome	4 (9.5)
Congestive heart failure	4 (9.5)
Infective endocarditis	3 (7.1)
Liver cirrhosis	3 (7.1)
Pneumonia	2 (4.8)
Interstitial pneumonia	2 (4.8)
Stroke	2 (4.8)
Others	6 (14.3)
Blood biochemical tests*	
Glucose, mg/dL	155 (125 to 227)
Lactate, mM	2.72 (1.92 to 5.78)
ATP, mM	0.38 (0.29 to 0.56)
A-LES	8.00 (4.74 to 13.37)
Hemoglobin, g/dL	10.4 (9.1 to 11.1)
Leukocyte count,/µL	12,700 (9,300 to 17,100)
Platelet count, x1,000/µL	114 (46 to 182)

Data are median (interquartile range) and number of patients (%).

* Data represent results of analysis of samples taken at admission to the ICU.

### Blood ATP Levels Normalized by Total Hemoglobin in ICU Patients

**Figure 1:**
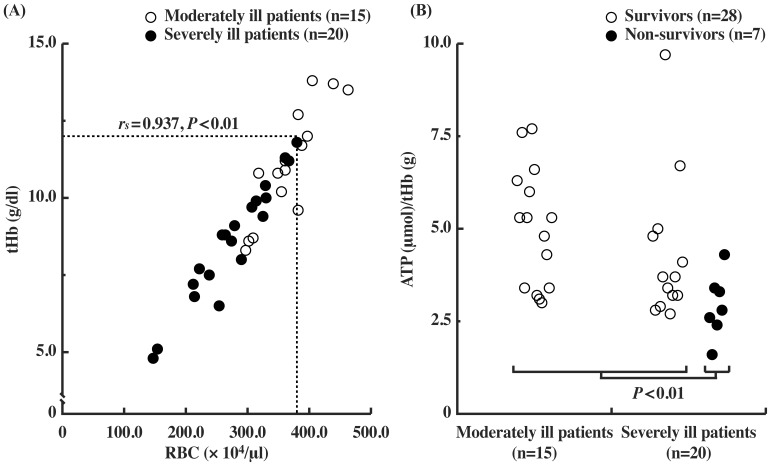
Blood ATP levels correlate with RBC count and ATP levels in ICU patients. (A) Correlation between tHb (total hemoglobin) and RBC count in the available data of moderately (n = 15) and severely ill patients (n = 20) during the ICU stay. The *Dotted lines* represent the lower limits of tHb and RBC count of healthy subjects. (○): moderately ill patients, (•) severely ill patients. (B) ATP concentration in whole blood, expressed as micromoles ATP per gram total hemoglobin (µmol/g tHb) in ICU patients. (○): survivors, (•) non-survivors. The values of ATP/tHb in non-survivors were significantly lower than those in survivors (*P*<0.01).

Blood cells are diluted by transfusion and low red blood cell (RBC) count is usually found in patients with advanced disease, resulting in low blood ATP levels in severely ill patients. Since the major source of ATP in blood is RBC, we determined RBC count and total hemoglobin (tHb) in moderately ill patients (APACHE II score <20) and severely ill patients (APACHE II score ≥20) during ICU admission. The RBC count correlated with tHb level (*r_s = _*0.937, *P<*0.01), and was significantly lower in severely ill patients than moderately ill patients (*P<*0.01) (Figure 1A). Blood ATP levels normalized by tHb levels (the ATP/tHb) were significantly lower in non-survivors than those in survivors (*P*<0.01) (Figure 1B).

### Changes in Blood ATP and A-LES Levels in Critically Ill Patients

**Figure 2:**
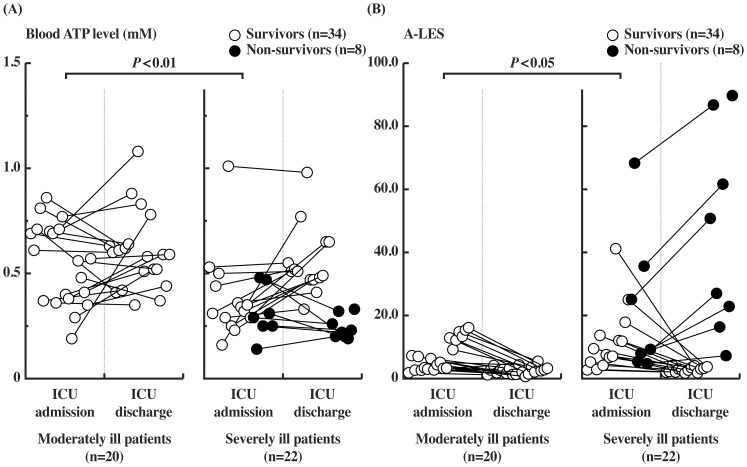
Changes in blood ATP and A-LES levels in moderately and severely ill patients. (A) Blood ATP levels and (B) A-LES values at ICU admission and ICU discharge. Symbols are paired data of individual patients. (○): survivors, (•) non-survivors. Levels of ATP and A-LES at ICU admission in severely ill patients were significantly lower than those in moderately ill patients (*P*<0.01 for ATP and *P*<0.05 for A-LES).

To identify a sensitive and real-time prognostic biomarker of critical illness, we evaluated the levels of ATP and A-LES in 42 patients during critical illness (Figure 2). ATP levels at ICU admission were significantly lower in severely ill patients than in moderately ill patients (*P*<0.01) (Figure 2A): the median ATP level was 0.31 mM (0.25 to 0.44) in severely ill patients and 0.56 mM (0.38 to 0.70) in moderately ill patients at ICU admission (Table 4). Notably, the median ATP level of 7 patients with septic shock on admission was low at 0.29 mM (0.27 to 0.33), which was significantly lower (*P*<0.01) than the level in moderately ill patients (Tables 4 and S2). The ATP levels generally recovered during ICU stay in large numbers of survivors. Furthermore, the median ATP level in non-survivors [0.23 mM (0.20 to 0.28)] was significantly lower than that of survivors [0.52 mM (0.45 to 0.62)] at ICU discharge (*P*<0.01) (Table 4).

**Table 4:** Comparison of changes in blood ATP level, A-LES, and APACHE II score during the course of ICU stay.

		ICU admission	ICU discharge
Moderately ill patients (*n = 20*)	ATP, mM	0.56 (0.38 to 0.70)	0.53 (0.44 to 0.61)
	A-LES	6.7 (3.1 to 12.4)	2.7 (1.8 to 3.2)
	APACHE II score	12.5 (9.0 to14.3)	11.5 (9.0 to 13.3)
Severely ill patients (*n = 22*)	ATP, mM	0.31 (0.25 to 0.44)**	0.45 (0.28 to 0.52) *
	A-LES	9.5 (6.8 to 17.9)*	3.6 (2.7 to 21.0) **
	APACHE II score	25.0 (20.0 to 31.0)**	21.0 (16.5 to 25.0)**
Septic shock patients (*n = 7*)	ATP, mM	0.29 (0.27 to 0.33)**	0.48 (0.33 to 0.57)
	A-LES	12.1 (10.7 to 19.4)*	3.1 (2.9 to 9.6) **
	APACHE II score	28.0 (22.0 to 36.0)**	15.0 (14.0 to 19.0)**
Survivors (*n = 34*)	ATP, mM	0.41 (0.33 to 0.60)	0.52 (0.45 to 0.62)
	A-LES	7.4 (3.7 to 12.7)	2.7 (2.1 to 3.3)
	APACHE II score	15.5 (11.3 to 22.3)	13.5 (11.0 to 16.0)
Non-survivors (*n = 8*)	ATP, mM	0.29 (0.25 to 0.39)#	0.23 (0.20 to 0.28)##
	A-LES	9.3 (6.7 to 30.4)#	38.9 (21.0 to 67.9)##
	APACHE II score	25.0 (20.0–31.0)##	21.0 (16.5 to 25.0)##
Total (*n = 42*)	ATP, mM	0.38 (0.29 to 0.56)	0.51 (0.36 to 0.59)
	A-LES	8.0 (4.7 to 13.4)	3.0 (2.1 to 4.2)
	APACHE II score	19.0 (13.0 to 25.0)	15.0 (12.3 to 21.5)

Data are median (interquartile range). *n* = number of healthy subjects.

* *P*<0.05,

** *P*<0.01 versus moderately ill patients: Mann-Whitney’s U-test.

# *P*<0.05,

## *P*<0.01 versus survivors: Mann-Whitney’s U-test.

In contrast to the changes in blood ATP levels during ICU admission, the change in A-LES was clearer particularly in severely ill patients and non-survivors (Figure 2B). The A-LES decreased in all survivors in both moderately and severely ill patients without exception and the median A-LES of survivors was 2.7 (2.1 to 3.3) at ICU discharge (Table 4). In contrast, the median A-LES in non-survivors at ICU discharge was 38.9 (21.0 to 67.9), which was significantly higher than the value of survivors (*P*<0.01) (Table 4). These results indicate that A-LES is a highly sensitive prognostic marker of critical illness.

### Evaluation of A-LES as a Prognostic Marker and Correlation with APACHE II in ICU Patients

**Figure 3:**
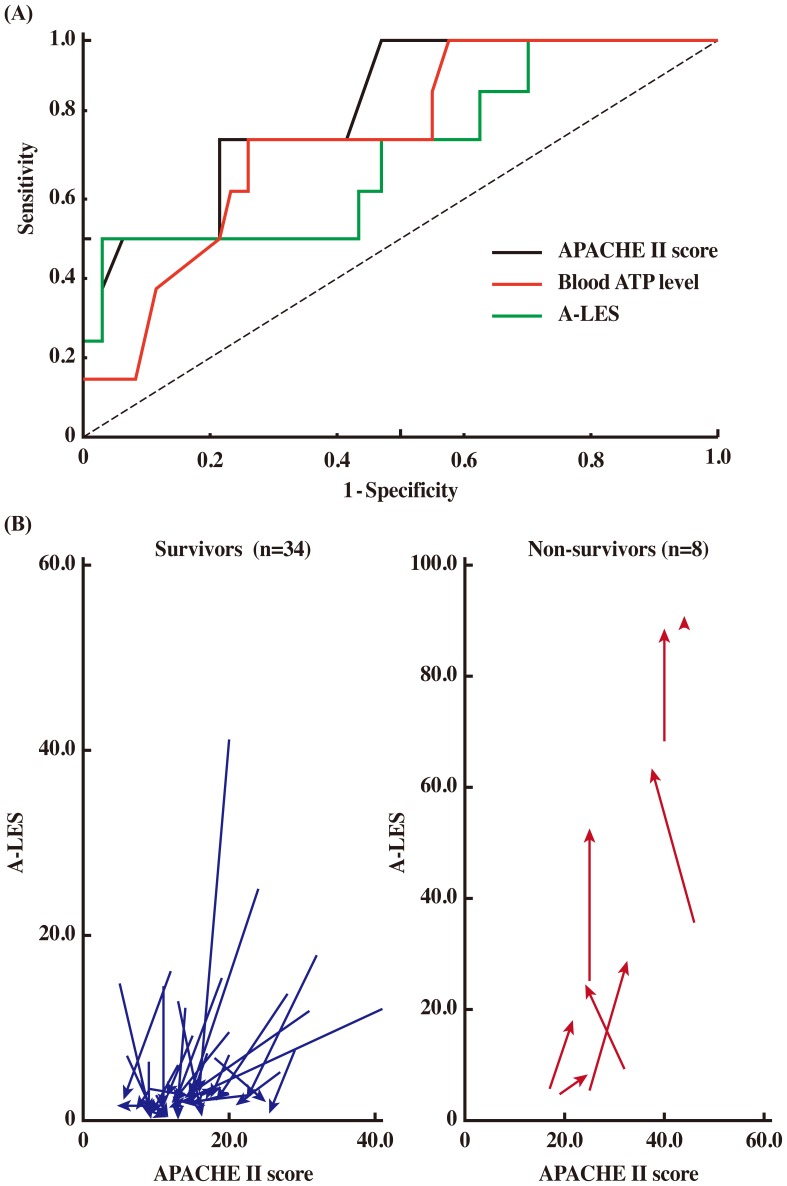
Relationship between APACHE II score and A-LES. (A) ROC analysis in prediction of mortality at the time of ICU admission. Dotted diagonal line = no discrimination. (B) Correlation between changes in APACHE II score and A-LES score during ICU stay in survivors and non-survivors. Data are values measured at two time points: at initial ICU admission and ICU discharge. Arrows indicate from ICU admission to discharge.

To evaluate A-LES and ATP levels of patients at the time of ICU admission for prediction of mortality, ROC analysis was performed (Figure 3A and Table 5). The values of the area under ROC curve (AUC) for APACHE II, ATP and A-LES were of similar range (>0.5) and measured 0.83, 0.75 and 0.71, respectively, indicating that ATP level and A-LES are as useful as APACHE II score for prediction of mortality.

**Table 5:** ROC analysis for prediction of mortality in 42 patients at ICU admission.

Variable	AUC	Cut-off value	Sensitivity/specificity (%)
APACHE II score	0.83	>20	71/59
A-LES	0.71	>20	43/94
Blood ATP levels*	0.75	>3	71/74

* The reciprocal of blood ATP levels was used for ROC analysis.

**Figure 4:**
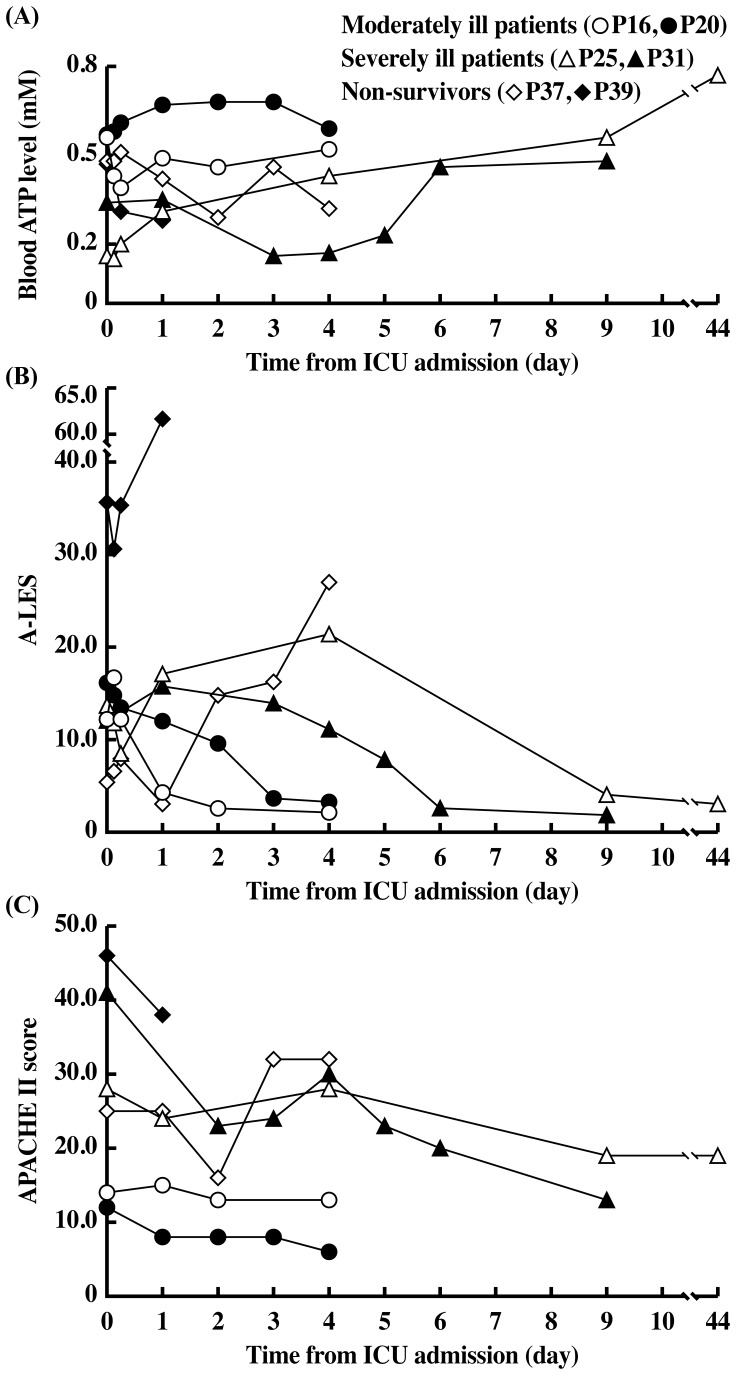
Changes in blood ATP level, A-LES, and APACHE II score of representative examples during the course of ICU stay. Serial changes in the levels of (A) blood ATP level, (B) A-LES, and (C) APACHE II score from admission to discharge from the ICU according to the severity of clinical condition (moderately and severely ill patients and non-survivors).

Figure 3B shows changes in A-LES and APACHE II scores measured during ICU admission. Although APACHE II scores did not sensitively express the change in the critical state of ICU patients, particularly patients with severe illness (APACHE II range, ≥20.0), A-LES reflected well the change in the critical state. Markedly high A-LES values (up to 89.7) in non-survivors and low values in all survivors were observed during ICU admission. These results indicate that A-LES does not only predict mortality at the time of ICU admission in a manner similar to APACHE II, but also provides a sensitive evaluation score of change in illness severity during ICU admission. Figure 4 illustrates the changes in blood ATP, A-LES and APACHE II during ICU admission of representative patients of the three groups (moderately ill patients on admission and discharge, severely ill patients on admission and discharge, and severely ill patients on admission who died during admission). Among the three parameters, A-LES provided the best prognostic information; almost all patients with satisfactory outcome at discharge had A-LES of <5.49, while A-LES during admission was >20.0 in non-survivors.

## Discussion

Risk prediction is an important issue in intensive care. The APACHE II score is used as a severity score during the first 24 hours of ICU admission while the SOFA score was developed to estimate morbidity during ICU stay. Although several clinical and laboratory parameters have been evaluated for the prediction of mortality during ICU stay, real-time and easily measurable prognostic biomarker(s) are desirable. During critical illness, the serum contains various PAMP molecules, particularly in severe infection [29], DAMP molecules released by stressed or damaged tissues [17] and host cellular response molecules with regulatory function against these PAMP and DAMP molecules. Among the molecules, alarm reporter(s) might be an important prognostic value. Recently, ATP released from damaged tissues has been classified as a danger signal, alarmin, which induces proinflammatory cytokines, but it is rapidly degraded within few minutes by ecto-ATPase [19]. Although released ATP cannot be analyzed accurately, cellular ATP levels, including blood cell ATP levels, can be measured easily, representing the sum of ATP production and ATP degradation and/or release.

We recently established an efficient and improved phenol-based ATP extraction method instead of the chaotropic extraction reagents recommended in the commercially available assay kit, which involves co-precipitation of ATP with insoluble proteins during homogenization and extraction [26]. In the present study, we measured blood ATP levels by a phenol-based extraction reagent and reported the “normal” blood ATP levels in 155 healthy individuals ranging in age from 0 to 92 years. The mean blood ATP level (SD) in healthy subjects was 0.62 (0.19) mM. Age and gender had no significant effect on ATP level, although the values tended to decrease with advancing age, particularly over 60 years of age, and values in males were slightly higher than those in females with some exceptions, probably because of age and gender differences in the number of red blood cells (Table 1).

The present study established the clinical utility of a sensitive and real-time alarm index, A-LES, which consists of ATP and lactate. The median A-LES of patients with satisfactory outcome at ICU discharge was 2.7 (2.1 to 3.3) and that of non-survivors was significantly high at 38.9 (21.0 to 67.9) (*P*<0.01) (Table 4). The A-LES was <20 in almost all moderately ill patients during ICU stay and was ≥20 in a large proportion of the non-survivors (Table 4 and Figure 3B). The results suggest that 20 is a critical cut-off value of A-LES for prediction of survival in the limited number of patients in this study. The change in A-LES ranged from 3.05 to 89.73 in non-survivors whereas the change in APACHE II score was ranged only from 17 to 46 (Table S3 and Figure 3B). These results suggest that A-LES provides better prognostic information compared to the APACHE II score. In addition to the value of A-LES during ICU admission, the AUC values for APACHE II, ATP and A-LES (Figure 3A) indicate that simple measurement of blood ATP and A-LES at the time of ICU admission predicts mortality in a manner similar to APACHE II, the complex and time-consuming evaluation method.

Although ATP released from damaged cells and tissues [17], [19] and from RBC in response to low PO_2_, low pH and/or mechanical deformation [30], [31] is an emergency signal alarmin, the levels in serum are difficult to monitor because the released ATP is rapidly degraded within few minutes [19]. The major source of ATP in the blood is the RBC and blood ATP levels change hourly with changes in energy and vital status of patients. Therefore, A-LES level is a real-time and sensitive biomarker of vital sign and a marker for prediction of mortality in critically ill ICU patients.

### Conclusions

This is the first report on blood ATP levels and A-LES as an alarm biomarker for critical illness during ICU stay and for the prediction of outcome of clinically ill patients at the time of ICU admission, similar to APACHE II score. In addition, A-LES provided further evaluation score of illness severity during ICU stay in addition to APACHE II particularly for those critically ill patients with a score of ≥20.0.

## Supporting Information

Table S1 Demographics and clinical details of moderately ill patients (APACHE II score<20). *Blood samples were collected at D0 = ICU day 0 (ICU admission), D1 = ICU day 1 (discharge or death from ICU within 24 hours) and D4 = ICU day 4 (discharge or death from ICU within 4 days). # Blood samples were collected from arterial blood (A) or central venous blood (CV). tHb = total hemoglobin; BS = blood sugar.
(DOC)

Table S2 Demographics and clinical details of severely ill patients (APACHE II score ≥20). *Blood samples were collected at D0 = ICU day 0 (ICU admission), D1 = ICU day 1 (discharge or death from ICU within 24 hours) and D4 = ICU day 4 (discharge or death from ICU within 4 days). # Blood samples were collected from arterial blood (A) or venous blood (V). For abbreviations, see Table S1.
(DOC)

Table S3 Demographics and clinical details of non-survivors. *Blood samples were collected at D0 = ICU day 0 (ICU admission), D1 = ICU day 1 (discharge or death from ICU within 24 hours) and D4 = ICU day 4 (discharge or death from ICU within 4 days). # Blood samples were collected from arterial blood (A). For abbreviations, see Table S1.
(DOC)
