# Supplementary material for: Blood Lactate/ATP Ratio, as an Alarm Index and Real-Time Biomarker in Critical Illness
Source: PLoS One. 2013 Apr 5;8(4):e60561. doi: 10.1371/journal.pone.0060561 (PMC3618266; doi:10.1371/journal.pone.0060561)
Supplement: Table S1 — Demographics and clinical details of moderately ill patients (APACHE II score<20). *Blood samples were collected at D0 = ICU day 0 (ICU admission), D1 = ICU day 1 (discharge or death from ICU within 24 hours) and D4 = ICU day 4 (discharge or death from ICU within 4 days). # Blood samples were collected from arterial blood (A) or central venous blood (CV). tHb = total hemoglobin; BS = blood sugar. (DOC) [file pone.0060561.s001.doc]

**Supporting Information**

**Blood Lactate/ATP Ratio, as an Alarm Index and Real-Time Biomarker in Critical Illness**

Junji Chida^1^, Rie Ono^2^, Kazuhiko Yamane^1^, Mineyoshi Hiyoshi^1^, Masaji Nishimura^2^, Mutsuo Onodera^2^, Emiko Nakataki^2^, Koichi Shichijo^3^, Masatami Matushita^3^, and Hiroshi Kido^1^

**Table S1. Demographics and clinical details of moderately ill patients (APACHE II score<20).**

| **Patients no.** | **Sex/Age** | **Diagnostic outcome** | **Time*/Vessel#** | **tHb (g/dl)** | **BS (mg/dl)** | **Lactate (mM)** | **ATP (mM)** | **A-LES** | **APACHE II score** |
| --- | --- | --- | --- | --- | --- | --- | --- | --- | --- |
| 01 | M/84 | Infective Endocarditis | D0/A | 11.0 | 330 | 1.52 | 0.69 | 2.20 | 12 |
|  |  |  | D1/A | 9.2 | 108 | 1.27 | 0.70 | 1.81 | 10 |
|  |  |  | D4/A | 9.3 | 174 | 0.71 | 0.38 | 1.87 | 15 |
| 02 | M/61 | Cerebellar Hemorrhage | D0/A | 11.5 | 136 | 4.43 | 0.61 | 7.26 | 17 |
|  |  |  | D1/A | 9.9 | 155 | 1.63 | 0.60 | 2.72 | 15 |
| 03 | F/34 | Pneumonia | D0/A | - | 115 | 1.91 | 0.71 | 2.69 | 16 |
|  |  |  | D1/A | 14.5 | 108 | 2.67 | 0.71 | 3.76 | 14 |
|  |  |  | D4/A | - | 91 | 1.98 | 0.35 | 5.66 | 14 |
| 04 | M/83 | Atrial Myxoma | D0/A | 10.7 | 212 | 5.67 | 0.81 | 7.00 | 6 |
|  |  |  | D1/A | - | 104 | 1.69 | 0.32 | 5.28 | 12 |
|  |  |  | D4/A | - | 162 | - | 0.36 | - | 7 |
| 05 | F/74 | Unstable Angina | D0/A | 11.0 | 93 | 0.98 | 0.37 | 2.65 | 9 |
|  |  |  | D1/A | 11.5 | 102 | 1.12 | 0.42 | 2.67 | 6 |
| 06 | M/37 | Pneumonia | D0/A | 16.3 | 189 | 2.95 | 0.86 | 3.43 | 9 |
|  |  |  | D4/A | - | 147 | 1.88 | 0.59 | 3.19 | 5 |
| 07 | F/81 | Aortic Stenosis, Angina | D0/A | 11.7 | 148 | 1.79 | 0.70 | 2.56 | 13 |
|  |  |  | D1/A | 8.2 | 136 | 1.68 | 0.71 | 2.37 | 12 |
|  |  |  | D4/A | - | 136 | 0.89 | 0.32 | 2.78 | 13 |
| 08 | M/61 | Acute Myocardial Infarction | D0/A | - | 226 | 6.43 | 0.57 | 11.28 | 13 |
|  |  |  | D1/A | - | 138 | 2.61 | 0.51 | 5.12 | 13 |
|  |  |  | D4/A | 13.4 | 137 | 1.49 | 1.24 | 1.20 | 8 |
| 09 | M/68 | Angina | D0/A | - | 176 | 2.29 | 0.36 | 6.36 | 9 |
|  |  |  | D1/A | - | 152 | 1.12 | 0.35 | 3.20 | - |
| 10 | F/75 | Congestive Heart Failure | D0/A | 9.2 | 227 | 1.95 | 0.71 | 2.75 | 10 |
|  |  |  | D1/A | 10.4 | 140 | 1.30 | 1.08 | 1.30 | 11 |
| 11 | F/18 | Infective Endocarditis | D0/A | 11.6 | 272 | 3.38 | 0.77 | 4.39 | 8 |
|  |  |  | D1/A | 10.4 | 124 | 1.12 | 0.83 | 1.35 | 9 |
| 12 | F/59 | Cerebellar Hemorrhage | D0/A | 12.7 | 135 | 2.06 | 0.40 | 5.15 | 15 |
|  |  |  | D1/A | 11.5 | 132 | 2.22 | 0.51 | 4.34 | - |
| 13 | M/61 | Congestive Heart Failure | D0/A | 12.4 | 94 | 1.23 | 0.38 | 3.24 | 9 |
|  |  |  | D1/A | 12.5 | 109 | 2.42 | 0.32 | 7.56 | 8 |
|  |  |  | D4/A | 12.8 | 282 | 1.20 | 0.58 | 2.07 | 8 |
| 14 | M/70 | Abdominal Aortic Aneurysm | D0/A | - | 124 | 2.45 | 0.19 | 12.89 | 13 |
|  |  |  | D1/A | - | 190 | 1.16 | 0.78 | 1.49 | 16 |
| 15 | F/71 | Perforation peritonitis | D0/A | 9.7 | 229 | 2.66 | 0.29 | 9.17 | 15 |
|  |  |  | D1/A | 7.0 | 144 | 1.87 | 0.52 | 3.60 | 12 |
| 16 | M/75 | Esophageal Cancer | D0/A | 11.7 | 236 | 6.82 | 0.56 | 12.20 | 14 |
|  |  |  | D1/A | - | 148 | 2.10 | 0.49 | 4.29 | 15 |
|  |  |  | D4/A | - | 143 | 1.11 | 0.52 | 2.13 | 13 |
| 17 | F/29 | Infective Endocarditis | D0/A | 11.3 | 134 | 7.11 | 0.48 | 14.81 | 5 |
|  |  |  | D1/A | 9.6 | 123 | 0.84 | 0.69 | 1.22 | 9 |
|  |  |  | D4/CV | - | 214 | 2.03 | 0.37 | 5.49 | 8 |
| 18 | M/65 | Aortic Regurgitation | D0/A | - | 235 | 6.10 | 0.42 | 14.52 | 11 |
|  |  |  | D1/A | 9.8 | 100 | 1.47 | 0.59 | 2.49 | 11 |
| 19 | F/83 | Tricuspid Regurgitation | D0/A | 10.2 | 242 | 5.39 | 0.35 | 15.40 | 19 |
|  |  |  | D1/A | 11.7 | 225 | 9.08 | 0.40 | 22.70 | 22 |
|  |  |  | D4/A | 8.7 | 126 | 1.53 | 0.39 | 3.92 | 14 |
| 20 | M/64 | Thoracic Aortic Aneurysm | D0/A | 10.8 | 207 | 9.20 | 0.57 | 16.14 | 12 |
|  |  |  | D1/A | 12.7 | 152 | 2.48 | 0.68 | 3.65 | 8 |
|  |  |  | D4/A | 13.8 | 145 | 1.93 | 0.59 | 3.27 | 6 |

* Blood samples were collected at D0=ICU day 0 (ICU admission), D1=ICU day 1 (discharge or death from ICU within 24 hours) and D4=ICU day 4 (discharge or death from ICU within 4 days).

# Blood samples were collected from arterial blood (A) or central venous blood (CV).

tHb=total hemoglobin; BS=blood sugar.
