# Supplementary material for: Blood Lactate/ATP Ratio, as an Alarm Index and Real-Time Biomarker in Critical Illness
Source: PLoS One. 2013 Apr 5;8(4):e60561. doi: 10.1371/journal.pone.0060561 (PMC3618266; doi:10.1371/journal.pone.0060561)
Supplement: Table S2 — Demographics and clinical details of severely ill patients (APACHE II score ≥20). *Blood samples were collected at D0 = ICU day 0 (ICU admission), D1 = ICU day 1 (discharge or death from ICU within 24 hours) and D4 = ICU day 4 (discharge or death from ICU within 4 days). # Blood samples were collected from arterial blood (A) or venous blood (V). For abbreviations, see Table S1. (DOC) [file pone.0060561.s002.doc]

**Supporting Information**

**Blood Lactate/ATP Ratio, as an Alarm Index and Real-Time Biomarker in Critical Illness**

Junji Chida^1^, Rie Ono^2^, Kazuhiko Yamane^1^, Mineyoshi Hiyoshi^1^, Masaji Nishimura^2^, Mutsuo Onodera^2^, Emiko Nakataki^2^, Koichi Shichijo^3^, Masatami Matushita^3^, and Hiroshi Kido^1^

**Table S2.** Demographics and clinical details of severely ill patients (APACHE II score ≥ 20).

| **Patients no.** | **Sex/Age** | **Diagnostic outcome** | **Time*/Vessel#** | **tHb (g/dl)** | **BS (mg/dl)** | **Lactate (mM)** | **ATP (mM)** | **A-LES** | **APACHE II score** |
| --- | --- | --- | --- | --- | --- | --- | --- | --- | --- |
| 21 | M/78 | Angina | D0/A | 11.0 | 125 | 1.47 | 0.53 | 2.77 | 23 |
|  |  |  | D1/A | - | 114 | 1.21 | 0.50 | 2.42 | - |
|  |  |  | D4/A | 9.6 | 121 | 1.13 | 0.55 | 2.05 | 15 |
| 22 | F/79 | Interstitial Pneumonia | D0/A | 11.1 | 151 | 1.62 | 0.31 | 5.23 | 27 |
|  |  |  | D1/A | 8.7 | 126 | 1.09 | 0.45 | 2.42 | 13 |
|  |  |  | D4/A | 7.8 | 168 | 1.16 | 0.42 | 2.76 | 14 |
| 23 | F/0 | Acute Subdural Hematoma | D0/A | - | 77 | 4.46 | 0.47 | 9.49 | 30 |
|  |  |  | D1/A | - | 86 | 0.98 | 0.42 | 2.33 | 19 |
|  |  |  | D4/A | - | 77 | 0.85 | 0.24 | 3.54 | 18 |
| 24 | F/68 | Congestive Heart Failure | D0/A | 10.1 | 171 | 1.43 | 0.50 | 2.86 | 20 |
|  |  |  | D1/A | 9.8 | 146 | 1.30 | 0.54 | 2.41 | 19 |
|  |  |  | D4/A | 9.6 | 112 | 1.06 | 0.51 | 2.08 | 16 |
| 25 | F/19 | Septic Shock | D0/A | 5.5 | 157 | 2.19 | 0.16 | 13.69 | 28 |
|  |  |  | D1/A | - | 120 | 5.30 | 0.31 | 17.10 | 24 |
|  |  |  | D4/A | 7.4 | 142 | 0.20 | 0.43 | 21.40 | 28 |
| 26 | M/83 | Septic Shock | D0/A | 7.8 | 136 | 2.77 | 0.29 | 9.55 | 20 |
|  |  |  | D1/A | 8.6 | 71 | 0.90 | 0.33 | 2.73 | 13 |
| 27 | M/71 | Acute Myocardial Infarction | D0/A | 10.4 | 411 | 7.67 | 1.01 | 7.59 | 29 |
|  |  |  | D1/A | 8.6 | 179 | 2.83 | 1.08 | 2.62 | 27 |
|  |  |  | D4/A | 8.8 | 129 | 1.72 | 0.98 | 1.76 | 26 |
| 28 | F/67 | Unstable Angina | D0/A | 7.4 | 197 | 1.69 | 0.25 | 6.76 | 20 |
|  |  |  | D1/A | 8.9 | 164 | 2.77 | 0.46 | 6.02 | 21 |
|  |  |  | D4/A | - | 182 | 1.30 | 0.53 | 2.45 | 20 |
| 29 | M/73 | Congestive Heart Failure | D0/A | 8.5 | 233 | 1.63 | 0.23 | 7.09 | 20 |
|  |  |  | D1/A | 8.2 | 175 | 1.74 | 0.31 | 5.61 | 17 |
|  |  |  | D4/A | 8.9 | 162 | 1.46 | 0.42 | 3.48 | 18 |
| 30 | M/66 | Acute Aortic Dissection | D0/A | 11.2 | 235 | 14.82 | 0.36 | 41.17 | 20 |
|  |  |  | D1/A | 13.7 | 145 | 2.43 | 0.54 | 4.50 | 19 |
|  |  |  | D4/A | 12.0 | 169 | 1.43 | 0.50 | 2.86 | 17 |
| 31 | M/74 | Septic Shock | D0/A | 9.1 | 119 | 4.10 | 0.34 | 12.06 | 41 |
|  |  |  | D1/A | 8.6 | 223 | 5.52 | 0.35 | 15.77 | 23 |
|  |  |  | D4/A | 9.9 | 132 | 2.23 | 0.16 | 13.94 | 24 |
| 32 | M/65 | Septic Shock | D0/A | 10.0 | 91 | 3.79 | 0.32 | 11.84 | 31 |
|  |  |  | D1/A | 10.0 | 96 | 2.85 | 0.36 | 7.92 | 31 |
|  |  |  | D4/A | 11.7 | 100 | 2.49 | 0.43 | 5.79 | 18 |
| 33 | M/55 | Alveolar Hemorrhage | D0/A | 4.8 | 105 | 6.25 | 0.32 | 17.89 | 32 |
|  |  |  | D1/A | 7.9 | 202 | 2.76 | 0.52 | 5.31 | 18 |
|  |  |  | D4/A | 9.7 | 165 | 2.13 | 0.65 | 3.26 | 23 |
| 34 | F/34 | Septic Shock | D0/A | 7.1 | 109 | 7.26 | 0.29 | 25.03 | 24 |
|  |  |  | D1/A | 10.0 | 176 | 4.43 | 0.47 | 9.43 | 18 |
|  |  |  | D4/A | 11.3 | 137 | 2.20 | 0.58 | 3.79 | 19 |

* Blood samples were collected at D0=ICU day 0 (ICU admission), D1=ICU day 1 (discharge or death from ICU within 24 hours) and D4=ICU day 4 (discharge or death from ICU within 4 days).

# Blood samples were collected from arterial blood (A) or venous blood (V).

For abbreviations, see Table S1.
