# Supplementary material for: Blood Lactate/ATP Ratio, as an Alarm Index and Real-Time Biomarker in Critical Illness
Source: PLoS One. 2013 Apr 5;8(4):e60561. doi: 10.1371/journal.pone.0060561 (PMC3618266; doi:10.1371/journal.pone.0060561)
Supplement: Table S3 — Demographics and clinical details of non-survivors. *Blood samples were collected at D0 = ICU day 0 (ICU admission), D1 = ICU day 1 (discharge or death from ICU within 24 hours) and D4 = ICU day 4 (discharge or death from ICU within 4 days). # Blood samples were collected from arterial blood (A). For abbreviations, see Table S1. (DOC) [file pone.0060561.s003.doc]

**Supporting Information**

**Blood Lactate/ATP Ratio, as an Alarm Index and Real-Time Biomarker in Critical Illness**

Junji Chida^1^, Rie Ono^2^, Kazuhiko Yamane^1^, Mineyoshi Hiyoshi^1^, Masaji Nishimura^2^, Mutsuo Onodera^2^, Emiko Nakataki^2^, Koichi Shichijo^3^, Masatami Matushita^3^, and Hiroshi Kido^1^

**Table S3. Demographics and clinical details of non-survivors.**

| **Patients no.** | **Sex/Age** | **Diagnostic outcome** | **Time*/Vessel#** | **tHb (g/dl)** | **BS (mg/dl)** | **Lactate (mM)** | **ATP (mM)** | **A-LES** | **APACHE II score** |
| --- | --- | --- | --- | --- | --- | --- | --- | --- | --- |
| 35 | F/61 | Pulmonary Thromboembolism | D0/A | 11.0 | 373 | 7.29 | 0.29 | 25.10 | 25 |
|  |  |  | D1/A | - | 544 | 13.20 | 0.26 | 50.77 | - |
| 36 | M/74 | Liver Cirrhosis | D0/A | 9.0 | 44 | 9.56 | 0.14 | 68.30 | 40 |
|  |  |  | D1/A | 6.8 | 30 | 17.35 | 0.20 | 86.75 | - |
| 37 | M/79 | Burn | D0/A | 14.3 | 305 | 2.59 | 0.48 | 5.40 | 25 |
|  |  |  | D1/A | 15.8 | 119 | 1.28 | 0.42 | 3.05 | - |
|  |  |  | D4/A | - | 244 | 8.69 | 0.32 | 27.00 | 32 |
| 38 | F/51 | Septic Shock | D0/A | 10.6 | 147 | 2.00 | 0.25 | 8.00 | 17 |
|  |  |  | D1/A | 10.3 | 180 | 3.76 | 0.40 | 9.40 | 20 |
|  |  |  | D4/A | - | 136 | 3.16 | 0.30 | 10.50 | 19 |
| 39 | F/72 | Liver Cirrhosis | D0/A | - | 79 | 16.75 | 0.47 | 35.64 | 46 |
|  |  |  | D1/A | 6.8 | 157 | 10.60 | 0.31 | 35.33 | 38 |
|  |  |  | D4/A | 6.5 | 94 | 12.33 | 0.20 | 61.65 | 38 |
| 40 | F/51 | Interstitial Pneumonia | D0/A | 9.5 | 131 | 1.47 | 0.31 | 4.74 | 19 |
|  |  |  | D1/A | 8.3 | 106 | 1.55 | 0.30 | 5.17 | 18 |
|  |  |  | D4/A | 8.0 | 137 | 1.50 | 0.33 | 4.55 | 27 |
| 41 | F/59 | Liver Cirrhosis | D0/A | 8.8 | 170 | 2.32 | 0.25 | 9.28 | 32 |
|  |  |  | D1/A | 8.8 | 95 | 2.39 | 0.24 | 9.96 | 26 |
|  |  |  | D4/A | 7.2 | 139 | 5.26 | 0.23 | 22.87 | 25 |
| 42 | M/72 | Septic Shock | D0/A | 7.7 | 160 | 29.61 | 0.33 | 89.73 | 44 |

* Blood samples were collected at D0=ICU day 0 (ICU admission), D1=ICU day 1 (discharge or death from ICU within 24 hours) and D4=ICU day 4 (discharge or death from ICU within 4 days).

# Blood samples were collected from arterial blood (A).

For abbreviations, see Table S1.
